# Anabolic Resistance of Muscle Protein Turnover Comes in Various Shapes and Sizes

**DOI:** 10.3389/fnut.2021.615849

**Published:** 2021-05-05

**Authors:** Kevin J. M. Paulussen, Colleen F. McKenna, Joseph W. Beals, Kenneth R. Wilund, Amadeo F. Salvador, Nicholas A. Burd

**Affiliations:** ^1^Department of Kinesiology and Community Health, University of Illinois at Urbana-Champaign, Urbana, IL, United States; ^2^Division of Nutritional Sciences, University of Illinois at Urbana-Champaign, Urbana, IL, United States; ^3^Center for Human Nutrition, Washington University School of Medicine, St. Louis, MO, United States

**Keywords:** sarcopenia, obesity, chronic kidney disease, dietary protein, exercise

## Abstract

Anabolic resistance is defined by a blunted stimulation of muscle protein synthesis rates (MPS) to common anabolic stimuli in skeletal muscle tissue such as dietary protein and exercise. Generally, MPS is the target of most exercise and feeding interventions as muscle protein breakdown rates seem to be less responsive to these stimuli. Ultimately, the blunted responsiveness of MPS to dietary protein and exercise underpins the loss of the amount and quality of skeletal muscle mass leading to decrements in physical performance in these populations. The increase of both habitual physical activity (including structured exercise that targets general fitness characteristics) and protein dense food ingestion are frontline strategies utilized to support muscle mass, performance, and health. In this paper, we discuss anabolic resistance as a common denominator underpinning muscle mass loss with aging, obesity, and other disease states. Namely, we discuss the fact that anabolic resistance exists as a dimmer switch, capable of varying from higher to lower levels of resistance, to the main anabolic stimuli of feeding and exercise depending on the population. Moreover, we review the evidence on whether increased physical activity and targeted exercise can be leveraged to restore the sensitivity of skeletal muscle tissue to dietary amino acids regardless of the population.

## Introduction

In healthy individuals aged ~18–50, who are not sedentary and eat sufficient daily amounts of protein and energy, the amount of skeletal muscle mass remains relatively unchanged throughout daily life (1). The maintenance of skeletal muscle mass is achieved by a daily net protein balance between muscle protein synthesis rates (MPS) and muscle protein breakdown rates (MPB). In contrast, skeletal muscle proteins are irreversibly lost with negatively imbalanced protein turnover, as observed with aging and catabolic pathologies. Muscle protein loss can occur as a result of increased MPB or reduced MPS (or by some combination of the two). However, it is believed that a desensitization of MPS to normal anabolic stimuli, such as feeding and exercise, may be the main culprit underpinning the age and disease-related decline in muscle quantity or quality (2, 3). This desensitization of MPS to normal anabolic stimuli, has also been shown in patients with kidney failure receiving maintenance hemodialysis (MHD), possibly due to their muscles being overstimulated (hyper-protein metabolic) from treatment (4). Finally, injury or illness leading to muscle disuse negatively influences post-absorptive MPS (5) and attenuates the postprandial anabolic response to protein ingestion in otherwise healthy young individuals (5, 6).

**Figure 1:**
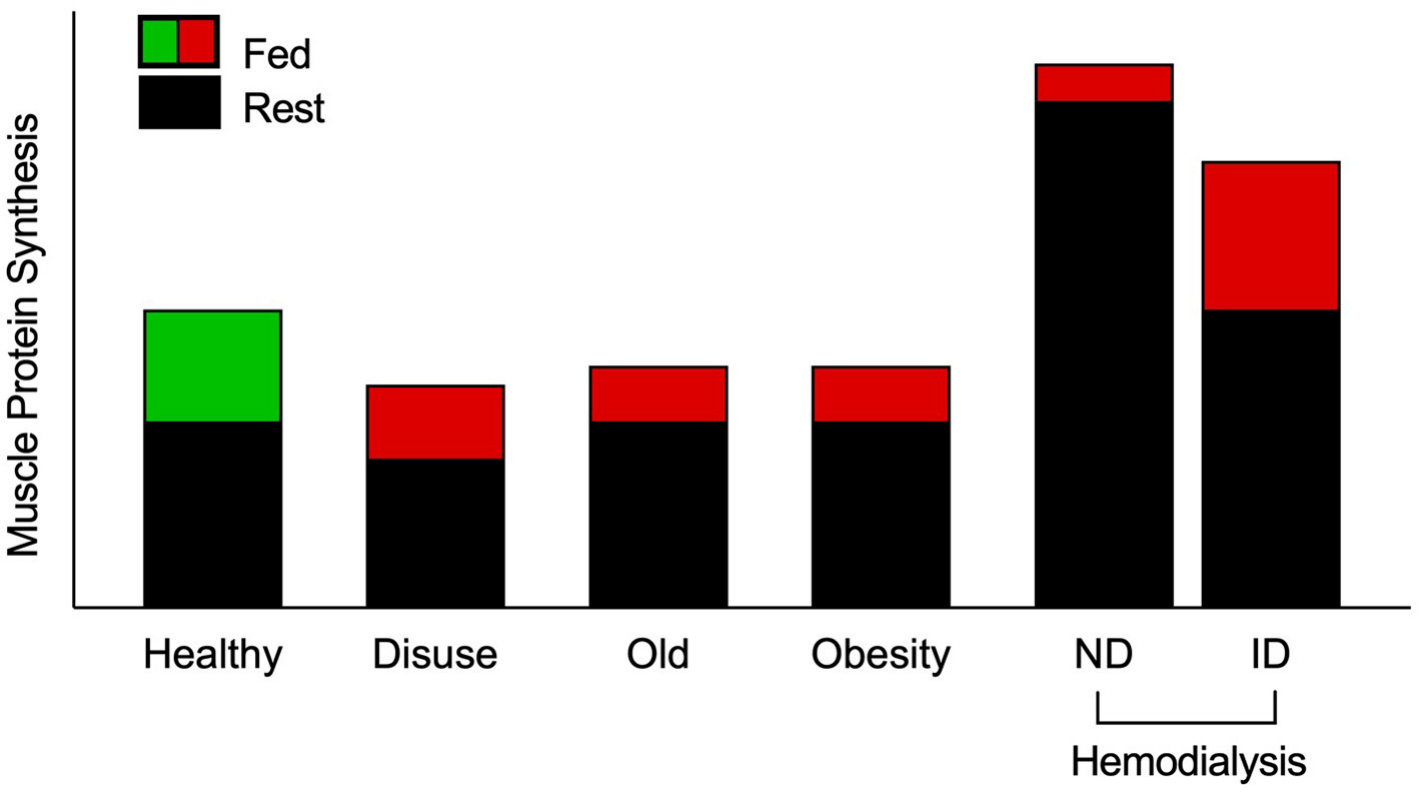
Schematic of muscle protein synthesis rates (MPS) in the basal and postprandial states. Eating a protein meal generally results in a ~2-fold increase in the stimulation of postprandial MPS from basal in healthy muscle. However, a reduced responsiveness of postprandial MPS, or anabolic resistance, to elevated plasma amino acid availability has been detected in older, obese, and adults on maintenance hemodialysis (MHD) (3, 4, 9–13). Muscle disuse is also a common facilitator of anabolic resistance. Hemodialysis patients have been studied on both dialysis and non-dialysis days. Despite peak inflammation (and presumably catabolism) during dialysis, MPS has been shown to be responsive to intravenous elevation of amino acid availability. However, skeletal muscle is overstimulated in the post-absorptive state on a non-dialysis day, which is associated with inflammation and uremic toxins, to induce overt anabolic resistance. Green indicates “healthy” fed-state MPS response, red indicates anabolically resistant fed-state MPS response. ND, non-dialysis day; ID, intra-dialytic.

It is evident that anabolic resistance has been detected in a number of conditions with different etiologies and is often regarded as the underlying cause of muscle mass loss. This ultimately affects physical performance and whole-body health given that the amount and quality of skeletal muscle regulates muscle strength and power, glucose disposal, fat oxidation, and energy balance (7, 8). As such, it is imperative to develop treatment strategies for anabolic resistance to minimize disease risk, retain independence, and ensure active involvement in family and community life. In this review, we discuss dietary and exercise considerations to both delay the onset of and minimize the negative consequences of anabolic resistance of muscle across pathologies. What is noteworthy is that the term “anabolic resistance” is often used to describe a decrease in the quality or quantity of a particular muscle protein pool, namely the myofibrillar proteins as opposed to the other protein sub-fractions (i.e., sarcoplasmic, mitochondrial, or collagen proteins). While anabolic resistance of myofibrillar protein synthesis rates is a common characteristic of aging and other compromised patient conditions, the underpinning mechanisms that are driving this poor responsiveness of contractile proteins to dietary amino acid availability in circulation are different between them. Therefore, the aim of this paper is to highlight the point that anabolic resistance manifests in different ways depending on the underlying etiology (Figure 1). We primarily focus on protein nutrition as this macronutrient has the strongest anabolic action on skeletal muscle tissue and, as such, appears to be the most susceptible macronutrient to anabolic resistance with aging and the various discussed clinical settings.

## Protein Turnover in Healthy Muscle

In healthy muscle, the consumption of isolated protein stimulates an increase of MPS in a dose-response manner (14, 15), with excess dietary amino acids being catabolized as opposed to utilized for postprandial MPS (15). The plateau of this dose-response curve has been shown to occur at ~0.25 g protein/kg body mass/meal in healthy young adults (9). The responsiveness of the postprandial muscle protein synthetic response is enhanced with the performance of resistance exercise, but not endurance exercise (16), prior to protein ingestion (17, 18). Based on the speed of amino acid absorption in relation to the stimulation of postprandial MPS, it has been shown that the ingestion of certain sources of isolated protein are more anabolic than others (e.g., whey > casein) (19, 20). Moreover, the amino acid composition of the ingested isolated protein source also has been shown to be an anabolic factor for the stimulation of MPS (20). In particular, the leucine content (21, 22), and the associated leucinemia (23), of the isolated protein source has been shown to potentiate postprandial MPS. Leucine is an anabolic signaling molecule acting through both mTORC1-dependent (24) and mTORC1-independent mechanisms (25) as well as a substrate to support the stimulation of MPS (26). While leucine serves both as a building block and signal, it is important to remember that all essential amino acids need to be available in order to maximize the anabolic potential of leucine on MPS (27, 28).

Historically, muscle protein metabolism investigations emphasize isolated sources of dietary protein, but it has become increasingly evident that protein foods are greater than the sum of their constituent amino acids for the regulation of postprandial MPS (29). This is likely due to food matrix (i.e., mixed macronutrients, micronutrients, and other bioactive molecules) effects facilitating a more anabolic molecular environment (30) and ultimately potentiating the use of dietary amino acids (including leucine) for postprandial MPS (31). Overall, whole protein foods provide the basis for healthy eating patterns with an option of ingesting isolated protein supplements to facilitate adequate total intake and assist with dietary protein distribution. The latter is believed to modulate daily net whole body (32) and muscle protein accretion (33).

In contrast to MPS, MPB appears to be less sensitive to changes in dietary amino acid availability. For example, MPB is elevated in the recovery period of both resistance and endurance exercise (34, 35), but these rates are not modifiable by dietary protein ingestion when compared to post-exercise MPS in healthy adults (18, 36). Therefore, nutritional strategies that target MPS, rather than MPB, can effectively achieve a desirable muscle protein balance (37) which additionally supports exercise training adaptations. Other factors such as eating below energy requirement (38), reduced daily carbohydrate intake (39), and habitually high protein intakes (40–42) can modulate dietary protein utilization, highlighting the value of achieving recommended nutrient intakes in relation to recommended energy intake (i.e., diet quality) to support optimal muscle adaptations. It is important to note that skeletal muscle is a heterogeneous tissue and is composed of a variety of different muscle protein pools such as myofibrillar, sarcoplasmic, mitochondrial, and collagen protein fractions. The myofibrillar proteins, which namely consist of actin and myosin, are the predominate protein pool within skeletal muscle tissue and account for ~60–65% of all muscle proteins. Myofibrillar proteins have a high nutrient sensitivity and thus have a substantial contribution to the stimulation of postprandial mixed MPS and preservation (or growth) of muscle mass throughout adult life (43, 44). Collectively, these observations provide the preliminary nutritional framework to support optimal skeletal muscle tissue anabolism. However, adverse alterations in muscle nutrient sensitivity evident with age, body adiposity, catabolic pathologies, and muscle injury or disuse, hinder the anabolic potency of dietary protein.

## Age-Related Anabolic Resistance

Several studies have shown no detectable differences in post-absorptive MPS between younger and older men (9, 45, 46) and women (47). It has been shown, however, that there is a decreased response of MPS to protein ingestion in older adults when compared to their younger counterparts (9, 45, 48). Recent work has shown a ~10% reduction in dietary protein digestion and absorption kinetics after protein ingestion with older vs. younger people (49), which likely contributes to age-related anabolic resistance of MPS (46). Such findings have been the impetus behind the development of strategies that target the postprandial period to attenuate age-related muscle mass loss as opposed to post-absorptive MPS. These dietary strategies have included meal fortification with free leucine (50), increasing the relative protein content of each meal (51, 52), manipulation of food texture (53), omega-3 supplementation (54), or a combination of various ingredients (55); all of which have shown promise for enhancing the acute postprandial MPS in aging muscle. Beyond these discrete, isolated meal manipulations, it is believed that daily meal frequency may also be an important variable that modulates 24 h net protein balance and, in extension, the maintenance of muscle mass with advancing age. For example, and similar to young muscle, a spread distribution pattern of protein intake (i.e., moderate, consistent amounts of protein at each meal) throughout the day is effective in maximizing the anabolic potential of each mealtime (33, 56). Considering this spread distribution pattern and higher protein requirement per meal, it seems that protein intakes for older adults are likely higher than the current recommended dietary allowance (RDA) in the United States or population reference intake (PRI) from the European Union, of ~0.8 g/kg/d and will be closer to ~1.2 g/kg/d (or higher). This notion is supported by previous studies using the indicator amino acid oxidation technique showing a safe intake of ~1.25 g/kg/d in adults aged 65 years and older (57). Another study observed that lean body mass loss over 3 years is lowest in older adults consuming ≥1.2 g/kg/d (58). All of this, however, is not completely surprising given that the protein RDA is set to meet the basic nutrient requirement as opposed to optimizing muscle mass with aging. Moreover, recent work has shown that the dietary leucine requirement for older adults is 78.5 mg·kg^−1^·day^−1^, which is more than double the RDA, currently established at 34 mg·kg^−1^·day^−1^ (59). This finding is pertinent as they suggest protein quality is likely another important factor for aging muscle. Specifically, higher quality food proteins are generally higher in leucine by total amino acid content and, as such, provide a more potent anabolic signal to skeletal muscle tissue. Overall, these observations show that dietary protein, and perhaps protein quality, can be a modifiable variable in diminishing the effects of anabolic resistance of MPS in an older population.

Age-associated anabolic resistance is not only associated with an impaired postprandial release of dietary amino acids into the circulation and a reduced responsiveness of MPS to protein ingestion, but it is also pervasive during recovery from both resistance- (60) and endurance-based exercise (61). Fortunately, exercise-specific strategies have been shown to effectively combat the age-related dietary amino acid insensitivity of MPS. For example, pre-conditioning strategies that enhance skeletal muscle capillarization (e.g., endurance training) prior to engaging in regular resistance exercise may be another strategy to enhance hypertrophic protein remodeling in older adults (62, 63). Also, there is some evidence to suggest that higher exercise volume might be associated with higher increases in lean body mass (60, 64, 65). While modification of the exercise prescription for an older adult may be necessary to robustly stimulate post-exercise MPS (66), it is clear that targeted exercise prior to protein ingestion improves aged-muscle nutrient sensitivity, leading to postprandial MPS comparable to young adults (17). For example, the anabolic effect of protein ingestion seems to be enhanced in older individuals as Pennings et al. showed that exercise performed prior to 20 g casein ingestion leads to no difference in post-exercise MPS between the young and old (17).

Ultimately, there appears to be a resistance to anabolic stimuli that potentially can be reversed by increasing doses of both protein ingestion (↑ anabolic signal) and prior exercise (↑ dietary amino acid sensitivity of muscle throughout recovery). Specifically, protein intake should be increased above the current RDA or PRI to be closer to ~1.2 g/kg/d. Exercise, resistance exercise in particular, seems to drastically increase the dietary amino acid sensitivity of older muscle to protein ingestion. Furthermore, as sedentary lifestyle behavior likely further exacerbates these effects, hence it is advisable to increase daily activity outside of leisure exercise.

Physical inactivity and the prolonged unloading of skeletal muscle tissue can play a significant role in the age-related loss of muscle mass. Indeed, sarcopenia is usually coupled with sedentary lifestyle behaviors during the aging process that can be attenuated with increased habitual physical activity (67, 68). In support, when assessing cumulative MPS in free living conditions by the use of deuterated water, Brook et al. demonstrated no differences in older individuals with higher-than-average activity levels when compared to young adults (69). When losing muscle mass at an older age, this is often further exacerbated by a diminished muscle re-growth in older individuals when compared to their younger counterparts after a period of muscle disuse (70) and going through these periods of “catabolic crises” may play a significant role in the loss of muscle mass during aging (71). The latter represents an active area of research whereby various strategies (e.g., neuromuscular stimulation, massage, etc.) are being tested and developed to attenuate muscle mass loss during injury/surgery that requires bedrest. Fortunately, it has been established that regular exercise at a more advanced age leads to an improvement of muscle protein remodeling by enhancing the postprandial MPS to protein ingestion. Hence, it is imperative for older adults to exercise (ideally incorporate some form of strength training) and adhere to healthy eating patterns (choosing nutrient-rich, whole foods) to stave off anabolic resistance as well as maximize skeletal muscle mass both in terms of quantity and quality to better handle an unforeseen catabolic crisis.

## Obesity-Related Anabolic Resistance

It is recognized that obesity, regardless of age, can impair the responsiveness of MPS to common anabolic stimuli (i.e., exercise and protein ingestion) compared with the response in sedentary healthy weight individuals (2, 3, 72–75). Indeed, several groups have attempted to investigate postprandial MPS in people with obesity with mixed results (2, 3, 72–78). Assessments of MPS to ingestion of protein-dense foods have shown that people with obesity have reduced stimulation of myofibrillar (3, 74), but not mitochondrial protein synthesis rates (76), compared with healthy-weight individuals. Similar to those experiments, work using intravenous delivery of amino acids (AA) and insulin also showed that people with obesity have lower rates of mixed muscle (72), myofibrillar (73), and mitochondrial (72) protein synthesis under those conditions when compared to non-obese controls. However, comparatively few studies have reported no differences in postprandial MPS between obese and control groups (75, 77, 78). Altogether, these studies indicate that postprandial stimulation of MPS is impaired in people with obesity and may relate to various metabolic changes (79). This impaired postprandial muscle protein remodeling with obesity likely impacts muscle metabolic quality as opposed to overall “bulk” muscle mass when considering lean body mass is generally the same (or greater) in obese vs. healthy weight individuals.

While older adults and obese individuals are both presented with anabolic resistance, the pathology behind this shared response (or lack thereof in more active aging muscle) is distinct between the two phenotypes. In direct contrast with aged muscle, whereby signaling protein content is lower than the young (45), obese individuals seem to have an overabundance of basal content and phosphorylation of intramuscular anabolic signaling proteins (e.g., mTORC1, p70S6K), which may uniquely inhibit the postprandial anabolic response in the presence of AA availability (2, 3, 75, 80). It is important to note that of the efforts thus far, none have directly compared muscle protein turnover with age-related sarcopenia, sarcopenic-obesity, and non-frail obesity. Of the obese individuals studied, the majority are young/middle-aged adults, with greater lean mass than their lean controls, and otherwise no mention of sarcopenia (2, 3, 72, 75–78). Similarly, investigations in older adults with obesity either observed comparable lean mass (74) or lack of evidence to classify as sarcopenic-obesity (73). More research is needed to understand the interplay between age-related sarcopenia and excess body adiposity on skeletal muscle regulation.

Outside the context of anabolic resistance, the frontline of obesity treatment involves lifestyle changes to improve body composition and reduce overall body mass, which requires achieving a negative energy balance through either dietary calorie restriction, increased energy expenditure, or a combination of these approaches. However, the loss of lean tissue with weight loss can undermine the maintenance or continuation of weight loss due to reduced metabolic rate (81, 82). Structured exercise is a fundamental strategy that simultaneously improves body composition and offsets weight loss-induced decrements in resting metabolic rate in people with obesity (82, 83). Exercise programs can largely be distinguished as endurance or resistance exercise training. In healthy young men, acute endurance exercise favors the stimulation of mitochondrial MPS during the postprandial period (16, 84), whereas resistance exercise tends to increase myofibrillar MPS (84). These findings suggest a targeted exercise prescription for individuals with obesity, given that anabolic resistance with obesity appears to specifically affect myofibrillar protein synthesis (2, 3, 73, 74, 77) more so than muscle mitochondrial protein synthesis (72, 75, 76).

The impact of acute resistance exercise on MPS and related anabolic signaling mechanism in people with obesity, however, is not clear (2, 85). One group observed no differences in fasted post-exercise MPS or anabolic signaling molecule phosphorylation (e.g., mTORC1) between obese and healthy-weight adults after a single bout of acute resistance exercise (85). More importantly, we have reported that a similar bout of resistance exercise did not augment the myofibrillar protein synthetic response or anabolic signaling after protein ingestion in obese participants compared to a healthy-weight control group (2). This observation further supports an apparent distinction between the anabolic resistance observed with aging and with obesity, as older individuals and young counterparts are conversely sensitized to the anabolic potential of dietary AA after resistance exercise (17).

While a mechanistic understanding of anabolic resistance to traditional stimuli warrants further investigation, interventional outcomes offer more conclusive recommendations. Ensuring an adequate intake of high-quality dietary protein (>1.2 g/kg/day) helps to preserve myofibrillar protein synthesis during pronounced short-term calorie restriction (~40% energy restriction) at rest (86, 87) and with resistance exercise in overweight or obese individuals (87). Additionally, the combination of dietary protein and resistance exercise may also be required for favorable metabolic adaptations to weight loss. Caloric-restriction investigations that manipulate dietary protein intake without concurrent exercise participation observe that weight loss-induced improvements in insulin-stimulated glucose disposal are blunted with higher protein diets (~1.2 g/kg/day) despite specific advantages of lean mass retention (88). These observations emphasize the need to consider both protein nutrition and intentional exercise when designing interventions to treat obesity.

Obesity is a variable condition with many phenotypes and is likely at the root of various observations discussed throughout this review. In all, the treatment of obesity is challenging and requires monitoring of not only body composition, but also co-morbid conditions that may negatively respond to some types of treatment. It is clear that weight loss alone can provide some metabolic benefit in terms of glucose metabolism, but whether long-term maintenance of weight lost and associated benefits requires greater protein intake needs more work, including longer-term interventions and follow-up. Nevertheless, it is clear that weight loss interventions should include an exercise component (with specific emphasis on resistance training) to alleviate lean body mass loss and increase dietary protein utilization.

## Anabolic Resistance With Catabolic Diseases

As previously described, there is a plethora of evidence to strongly support that the insidious pathology of aging or excess adiposity both result in the anabolic resistance of skeletal muscle. Therefore, it is unsurprising that more overt perturbations in human health also negatively impact muscle response to integral stimuli that support metabolic and physical capacity. Individuals with terminal diagnoses often exhibit aberrant and relentless (i.e., unresponsive to clinical nutrition support) muscle wasting, which is associated with poor quality of life and increased mortality (89).

Cancer, for example, may progress to a cachectic state that is characterized by pervasive catabolism and irreversible losses of muscle mass (90). Hence, there is a clear disruption of net protein balance that likely results from a reduced post-absorptive MPS (91), elevated MPB (92), and/or a postprandial anabolic resistance of MPS (92). Despite this anabolic resistance of fed-state MPS with cancer cachexia, targeted dietary support may show promise in this population. For example, a high-protein (40 g) nutritional beverage has been shown to overcome anabolic resistance when compared to the ingestion of a standard supplement (24 g casein protein) in cancer cachexia patients (93). In addition to a higher protein content (24 g casein fortified with whey protein, free amino acids, and free leucine) than control, this particular treatment beverage also contained EPA and DHA. These omega-3 fatty acids have been shown to enhance MPS (54). Indeed, further research is needed to delineate the contributions of each bioactive constituent to the stimulated postprandial MPS and ultimately for the development of a more anabolic clinical feeding formula. Furthermore, no empirical data to date exist on the magnitude and potential amelioration of anabolic resistance of MPS in cachectic cancer patients.

Muscle wasting is not only observed in malignant disease states but is also present with organ failure, most notably renal failure. End-stage renal disease (ESRD) is often a result of chronic disease (e.g., hypertension, diabetes) progression, thus accompanied by other co-morbid pathologies. In the absence of a kidney transplant, ESRD patients require chronic dialysis, typically in the form of intermittent (i.e., every 48–72 h) hemodialysis (MHD) therapy, to survive. MHD is an imperfect treatment, particularly in the context of protein metabolism, as one treatment session removes up to 15 g of circulating amino acids (94). This observation supports previous efforts that showed a net negative protein balance in skeletal muscle with MPB exceeding MPS after MHD treatment (95, 96). Intradialytic feeding is a therapeutic opportunity to combat these observed imbalances in nutrient handling and net losses of functional body mass in MHD patients. Both intradialytic parental nutrition (IDPN) (97) and oral supplemental nutrition (ONS) (10, 98) have been shown to successfully improve plasma amino acid availability and shift acute whole-body and muscle protein balance from negative to positive. However, feeding-induced positive protein balance is sustained in the post-MHD phase only with oral administration (10). Clinical relevance of these acute observations has been investigated: ONS is associated with reduced mortality in retrospective analyses (99–101), and improved serum albumin and prealbumin in a prospective investigation (102). However, these longitudinal analyses are not without limitations, including treatment adherence (compliance affecting feasibility) and the intrinsic flaws of measured parameters like BMI (does not account for lean mass) and serum albumin (negative acute-phase protein). Conversely, investigations outside of the dialysis treatment window may provide insight on the potential of targeted exercise and feeding strategies on the stimulation of muscle protein accretion in individuals with ESRD on MHD, especially given MHD patients spend the majority of their time outside the dialysis clinic. Interestingly, we have shown the hyper-stimulation of post-absorptive myofibrillar protein synthesis rates, which is driven by enhanced MPB, in MHD patients on non-dialysis days in comparison to age- and BMI-matched controls (4). Furthermore, the ingestion of a mixed meal, which contained ~20 g protein, resulted in impaired postprandial release of dietary leucine into the circulation, which was insufficient to induce stimulation of the postprandial myofibrillar protein synthetic response in MHD patients, despite adequately stimulating MPS in controls. Hence, there appears to be a two-pronged problem contributing to anabolic resistance in patients receiving dialysis treatment (i.e., hyper-stimulated basal muscle protein turnover and impaired protein digestion/absorption kinetics). Nonetheless, an unresolved question arises from this work: how do you fix it? We believe a logical starting point is to reduce the inflammatory and/or uremic milieu to ultimately reduce basal muscle protein turnover in MHD patients (103). The latter will perhaps allow for dietary amino acids to properly act as anabolic signaling molecules and substrates for the stimulation of postprandial MPS in individuals on MHD. This anabolic resistance related to the over stimulation of basal muscle protein turnover is likely underpinning the ineffectiveness of targeted exercise strategies in this patient population as well (104). In fact, Draicchio et al. (105) also demonstrated that there are clear disruptions in proteins associated with the extracellular matrix in patients on MHD, which likely is another contributing factor to the poor mechanotransduction of exercise signals to the muscle protein synthetic machinery. There is likely some level of anabolic resistance in the muscle of even the healthiest of MHD patients, and this is likely exacerbated in patients with overt wasting or sarcopenic obesity. So, it is critically important to attenuate muscle wasting in as many MHD patients as possible in order to maintain an adequate quality of life while in dialysis, to keep patients healthy enough to maintain transplant eligibility, and to improve prognosis following transplant. Importantly, the nephrology field has acknowledged the current short-comings of exercise prescription for MHD patients and have developed working groups (e.g., the Global Renal Exercise Working Group [GREX]) (106) to provide a sounding board for researchers in this field and, as such, improve the efficacy of exercise and nutritional interventions in this patient group.

## Physical Inactivity and Muscle Disuse: a Common Denominator

The old adage of “use it or lose it” is blatantly accurate as it relates to muscle mass loss with disuse regardless of age or disease state. Specifically, the term disuse describes a spectrum of behavior from reduced habitual physical activity to whole-body disuse. This includes complete disuse, like immobilization or bedrest, which can lead to a decrease in skeletal muscle mass and strength. But also more modest reductions in physical activity that can lead to decrease nutrient sensitivity and a lower muscle anabolic response (67).

It has been well-established that a prolonged period of disuse can lead to a rapid loss of total skeletal muscle mass and strength (107). More recently, it has also been shown that a period of short-term disuse can lead to dramatic losses of skeletal muscle mass and strength (11). Van Loon's research group observed that 2 weeks of one-legged immobilization impaired the MPS response to the ingestion of 20 g protein in healthy young men, and this led to an ~8.5% decrease in muscle CSA (=350 g of lean tissue) (11). This resembles previous studies that show a general atrophy rate of 0.5–0.6% per day during a short period of disuse (108). In the study, 2 weeks of disuse led to a ~25% loss of muscle strength, rapidly decreasing both muscle mass and quality. Looking at even shorter periods of disuse, the same research group has shown that after 5 days of single leg immobilization subjects lost 3.9 ± 0.6% CSA of the immobilized leg when compared to the control leg. Furthermore, the researchers showed a decrease in post-absorptive MPS and, more interestingly, despite an increase in circulating plasma insulin and leucine, a decrease in post-prandial MPS in all participants (109). More recently, it has been confirmed that this decreased MPS is sustained throughout the day in free-living conditions during a 7-day period of disuse (110). Hence, a short period of disuse can lead to a rapid onset of anabolic resistance in otherwise healthy young men.

While a complete cessation of movement has a considerable impact on skeletal muscle mass and strength, even a decrease of daily activity can have substantial effects on the anabolic capacity of the muscle. For example, 2 weeks of reduced activity reduces anabolic sensitivity to protein ingestion as observed by Breen et al. (67). In this study, otherwise healthy older adults reduced their daily step-count by ~76% to 1,413 ± 110 steps per day. Postprandial insulin sensitivity decreased by ~43% and post-absorptive insulin resistance increased by ~12%. Postprandial MPS decreased by ~26% after the intervention, whereas post-absorptive MPS stayed the same. In comparison, the study from van Loon's group used a model whereby the volunteers received a full leg cast for the same time period of 2 weeks. After the study period, postprandial mixed MPS decreased by ~31%. Additional work on a reduction of physical activity was done by McGlory and colleagues (111). In this study, the researchers confirmed that 2 weeks of step reduction leads to a decrease in insulin sensitivity and an increase in insulin resistance. In addition, McGlory et al. (111) provided an important extension to these findings showing a ~35% decrease in estimated insulin sensitivity (i.e., Matsuda insulin sensitivity index) and a 23% increase in insulin resistance derived from HOMA-IR. More importantly, these indices of insulin sensitivity/resistance were not restored after 2 weeks of return to habitual physical activity.

Obesity (112), aging (113, 114), and chronic illnesses (114) are all associated with a decrease in the number of steps taken per day and an increase in sedentary time. Concurrently, in a study by Smeuninx et al., older obese and lean individuals showed a significant positive correlation (r^2^ = 0.33) between average daily step count and the net postprandial myofibrillar protein synthetic response (74). Mutual mechanisms underpin the hypothesis that physical inactivity is a critical component of anabolic resistance. The master regulator for MPS mammalian target of rapamycin complex 1 (mTORC1) is clearly reduced during decreases in physical activity, as evidenced by Shad et al. (115) and in line with increased myostatin expression after complete muscle disuse (109). Comparatively, phosphorylation of mTORC1 does not occur in older individuals up to 24 h after exercise and food ingestion when compared to their younger counterparts (116). The more downstream target, eukaryotic translation initiation factor 4E-binding protein 1 (4E-BP1), showed higher phosphorylation levels after ingesting a meal in habitually active individuals in a study done by Breen et al. (67). In contrast, after 2 weeks of step-reduction those same individuals failed to show an increase in 4E-BP1 activation. Comparatively, our lab has shown an inability to phosphorylate 4E-BP1 after exercise in obese individuals when compared to normal weight individuals (2). Similarly, others have demonstrated that phosphorylation of 4E-BP1 does not occur in older individuals up to 24 h after exercise and food ingestion when compared to their younger counterparts (116).

Skeletal muscle insensitivity to the normally stimulatory effects of dietary amino acids in circulation and exercise contributes significantly to muscle disuse atrophy. This anabolic resistance plays an overt role in the development of age-related sarcopenia and obesity. The commonality of decreased physical activity/muscle contraction between these conditions is certainly a contributing factor for the associated anabolic resistance. It is still not clear, however, how various forms of sedentary behavior impact anabolic resistance. For example, sedentary behavior refers to low levels of energy expenditure ( ≤ 1.5 METS) during waking behaviors such as sitting, reclining, or lying (117), and is different from being physically inactive. In other words, individuals can be largely sedentary (i.e., sitting ≥8 h/d) (118), but meet the minimal amount of recommended physical activity per week (117). It seems reasonable to assume that breaking up prolonged sedentary blocks of time with so-called “exercise snacks” is likely beneficial for minimizing lower levels of anabolic resistance when compared to experiencing too much sedentary time together throughout the day (119). Moreover, certain populations (such as MHD patients) have a higher degree of anabolic resistance that cannot be simply solved by prescribing more physical activity and exercise as discussed previously (103). Instead, this compromised patient condition will require a more comprehensive lifestyle intervention strategy and individualized exercise prescription to overcome MHD-related anabolic resistance (104).

## Conclusion

A central message of this paper has been that anabolic resistance is a common factor underpinning muscle mass loss with aging and other compromised disease states. In an acute setting, targeted exercise prior to protein ingestion has been shown to enhance the dietary amino acid sensitivity of aging muscle (17), and thus is a clear fountain of youth for muscle. The anabolic resistance of myofibrillar protein synthesis rates with obesity (3), at least in our hands, cannot be completely abolished with resistance exercise prior to food intake (2). Instead, we have shown that the dietary amino acid remained confined to the more rapidly turning over sarcoplasmic pool in the post-exercise state (2). Certain catabolic diseases, such as MHD, demonstrate a unique form of anabolic resistance when compared to obesity or aging (4). Specifically, post-absorptive myofibrillar protein synthesis rates are elevated in MHD patient's muscle and create a stimulatory MPS ceiling effect such that dietary amino acids lose their anabolic signaling potential (4). This MHD-related hyper-stimulation of post-absorptive muscle protein turnover likely stems from the accumulation of the inflammatory and/or uremic milieu. As such, we speculate that this catabolic milieu will necessitate a dampening before the effectiveness of targeted exercise and feeding strategies can be observed in MHD patients (120).

**Figure 2:**
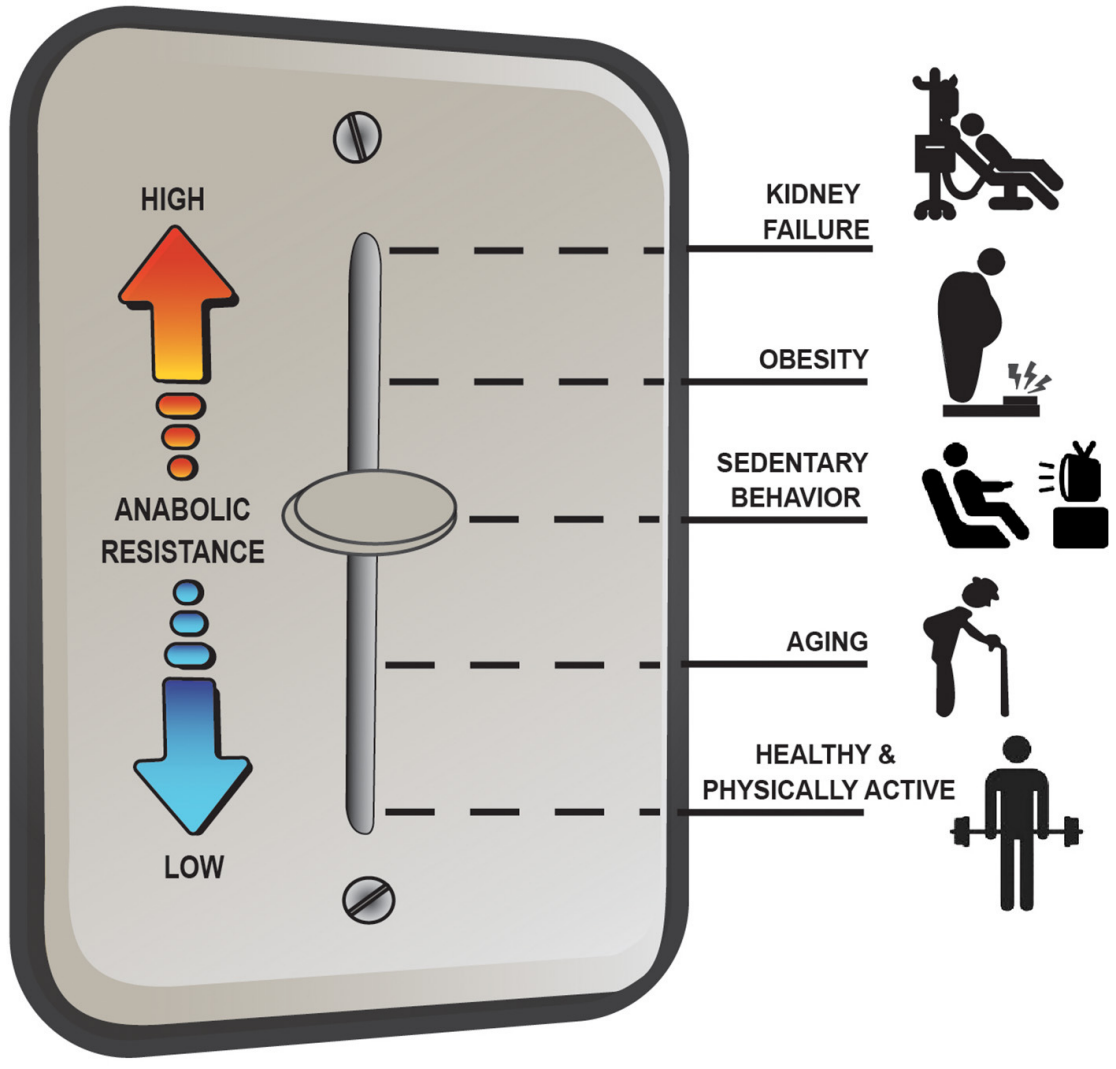
Anabolic resistance of muscle protein synthesis rates (MPS) to dietary amino acids exists as a dimmer switch from low to higher levels. Healthy aging muscle has been shown to be quite responsive to the combined stimuli of feeding and exercise in terms of an MPS response (17), but in absence of prior exercise anabolic resistance is detectable (46). Decreasing the physical activity level, which is perhaps exacerbated further when combining increased sedentary behaviors, increases the level of anabolic resistance on MPS (67). Similarly, obesity increases the level of anabolic resistance, regardless of age (3, 74), with the highest level of anabolic resistance on MPS existing in highly catabolic conditions such as end-stage renal disease (4).

Overall, the anabolic resistance of myofibrillar protein synthesis rates comes in various forms and can differ depending on the population studied. Basic advice such as moving more and eating more protein above the RDA to target the stimulation of post-exercise/postprandial MPS is effective under most situations of anabolic resistance; however, it is not a universal remedy for all situations (e.g., MHD muscle). Instead, there seems to be more of a dimmer switch (Figure 2) for anabolic resistance whereby the level it manifests depends on various factors including habitual physical activity level and dietary patterns, adiposity, and other metabolic abnormalities. For example, aging muscle can remain quite responsive to anabolic signals provided healthy lifestyle strategies are employed (i.e., a lower level of anabolic resistance) whereas MHD muscle is over stimulated at baseline and unresponsive to postprandial (and likely post-exercise) anabolic signals (i.e., a high level of anabolic resistance) (17). Research initiatives, such as the NIH Precision Nutrition and the Molecular Transducers of Physical Activity Consortium, will ideally provide better insight into individualized dietary and exercise recommendations to optimize health and quality of life throughout the lifespan and, as such, reduce the prevalence and/or severity of anabolic resistance.

## Author Contributions

NB, CM, JB, KW, and KP drafted the manuscript. KP and AS prepared the figures. All authors contributed to manuscript revision, read, and approved the submitted version.

## Conflict of Interest

NB has received research grants, consulting fees, and speaking honoraria from Dairy Management Inc. (DMI), the National Cattlemen's Beef Association, and Alliance for Potato Research and Education (APRE). This work was supported, in part, by the NIH training grant T32 HL130357 to JB. The remaining authors declare that the research was conducted in the absence of any commercial or financial relationships that could be construed as a potential conflict of interest.
